# Method of variability optimization in pharmacokinetic data analysis

**DOI:** 10.1007/s13318-013-0145-x

**Published:** 2013-06-19

**Authors:** Tomasz Grabowski, Jerzy Jan Jaroszewski, Walerian Piotrowski, Małgorzta Sasinowska-Motyl

**Affiliations:** 1 Polpharma Biologics, Trzy lipy 3, 80-172 Gdańsk, Poland; 2 Department of Pharmacology and Toxicology, Faculty of Veterinary Medicine, University of Warmia and Mazury, Oczapowskiego 13, 10-718 Olsztyn, Poland; 3 Biostatistical Laboratory, Department of Epidemiology, Cardiovascular Disease Prevention and Health Promotion Institute of Cardiology, Warsaw, Poland; 4 Departmentof Pharmacodynamics, Medical University of Warsaw, Krakowskie Przedmieście 26/28, 00-927 Warsaw, Poland

**Keywords:** Standard deviation, Sampling, Variability, Pharmacokinetics

## Abstract

For many drugs administered *per os*, high variability in the concentration–time (C–T) values from first sampling to the phase of distribution may cause difficulty in pharmacokinetic analysis. Therefore, the aim of this study was to propose a method of transformation of C–T data, which would allow significantly reducing the standard deviation (SD) value of observed concentrations, without a statistically significant influence on the value of the mean for each sampling point in group. In the presented study, the lowest value of relative standard deviation of concentrations observed in the elimination phase and the value of precision of the used analytical method, were used to optimize the arithmetic, geometric means, median, and the value of SD obtained after single oral administration of itraconazole in human subjects. Non-compartmental modeling was used to estimate pharmacokinetic parameters. The analysis of SD pharmacokinetic parameters after C–T value optimization indicated more than twice the lower value of SD. After transforming the itraconazole data, lower variability of concentration data gives more selective pharmacokinetics profile in absorption and early distribution phase.

## Introduction

The arithmetic, geometric and harmonic means, as well as the standard deviation (SD), as the measures of variability, are the ones most frequently used descriptive statistics in the calculation of pharmacokinetic (PK) (Cocchetto et al. 1980; Lam et al. 1985; Roe and Karol 1997; Griffin et al. 1999; Koch 1985; Julious and Debarnot 2000). A common problem in the analysis and interpretation of comparative pharmacokinetic parameters is the high value of SD (Davit et al. 2008; Haidar et al. 2008; Van Peer 2010). In extreme cases, the high value of SD makes it impossible to make the right decision as to the fate of the study. This problem concerns many types of studies, from preclinical studies, pilot pharmacokinetic studies to bioequivalence (BE) (Riley 2001; Chien et al. 2005). One way to solve the problem of comparative analysis of data burdened with high values of SD is to optimize the sample size of the group in the study, which consists of a precise determination of the number of subjects or animals on the basis of intrasubject variability (FDA 2006; Ramirez et al. 2008; EMA 2010). This is usually possible for BE studies. However, even in BE studies, in some cases, to determine the correct number of subjects, a pilot study is needed or even a two-stage study model (EMA 2010). A high value of variability (CV %) of observed concentrations or PK parameters, especially often makes it impossible to properly interpret the outcome of the study in case of research concerning new drugs, for which their value of variability of key PK parameters is unknown. This situation is further complicated by the fact that research in the early stages of drug discovery and pilot studies is usually conducted on a small number of subjects (for example first in man).

The scatter of results described by mean values and SD is the result of many factors. To a large extent it depends on the fate of the drug in the organism, such as absorption, distribution, re-distribution, metabolism and elimination. One of the factors standing “outside” of a living organism is the scatter of results, which comes from the precision of the used analytical method. Currently, the limit value for the CV % of precision for the calibration curve excluding the lower limit of quantitation point (LLOQ) is 15 %, while in the point equal to LLOQ, this value can be ≤20 % (FDA 2001). In relation to incurred sample analysis for classic drugs, as the acceptable range of differences for repeated analysis, the range 20 % is proposed (EMA 2009; Rozet et al. 2011; Yadav and Shrivastav 2011). This means that every bioanalytical result introduces an error to the pharmacokinetic calculations as well as the chosen research model—human, laboratory animal or cells in the in vitro studies (Jansen et al. 2002; Jones 2009).

For many drugs, the problem of analysis of PK after *per os* administration of the drug is high variability in the C–T values from first sampling to the phase of distribution. The common factor influencing maximum concentration (*C*_max_) and last concentration (*C*_last_) values is the spread of analysis result, which determines the precision of the analytical method. All three, absorption, distribution and elimination, processes which in point of time corresponding to *C*_max_ occur simultaneously. In case of a single administration of the drug in the elimination phase, the values of the concentration can be observed, which illustrate almost exclusively elimination processes (excluding the redistribution phenomenon), until the interval between the end of the distribution phase and the value equal to $t_{\hbox{max} } + t_{1/2kel} \times 3$ (EMEA 2001; Veng-Pedersen 2001; FDA 2006; HC 2010). It can be assumed that the factors that affect the *C*_max_ relative standard deviation (*C*_max, CV %_), resulting from simultaneously occurring elimination, are to some extend dependent on the value of last concentration relative standard deviation (C_last,CV %_) minus the error resulting from the precision of the analytical method (CV_ %,an_).

PK studies are usually conducted in the conditions of good laboratory practice and good clinical practice, or in accordance with the principles of these quality systems. It can be therefore assumed that the sum of errors connected to the subject, experimental animal, used formulation or bioanalytical method is constant, while keeping the experiment conditions, controlled by the quality system. It can also be assumed that in each PK study, a minimum range of SD for C–T is possible to achieve. In relation to a single administration, the closest value in many cases could be the lowest value of CV % for the last points of sampling in the elimination phase. In this phase of the study, the deviations from the mean are usually the lowest in the whole series, as the elimination phase is the dominant one and no other process, which is characterized by high variability (for example absorption) influences the SD of the analyzed concentrations. Taking the above into account, the aim of this study was to propose a method of transformation of C–T, which would allow significantly reducing the SD value of observed concentrations, without the statistically significant influence on the value of the mean and median for each sampling point.

## Materials and methods

### Pharmacokinetic data

In the presented study, the lowest value of relative standard deviation (RSD %) of concentrations observed in the elimination phase and the value of precision of the used analytical method were used to optimize the arithmetic and geometric mean and the value of SD obtained after single oral administration of itraconazole, which is characterized by high variability of pharmacokinetic parameters. A single dose of 100 mg of itraconazole was administered orally (Sporanox^®^ 100 mg tab., Janssen Pharmaceuticals) for male subjects ≥20 to ≤40 years old, with a body mass index ≥20 to ≤25 kg/m^2^. Blood samples were collected just prior to administration and at 0.5, 1.0, 1.5, 2.0, 2.5, 3.0, 3.5, 4.0, 4.5, 5.0, 6.0, 8.0, 12.0, 24.0, 36.0, 48.0, and 72.0 h after the administration. The concentration analyses were performed using tandem mass spectrometry, using the method described previously (Grabowski et al. 2009). The study was approved by Independent Ethics Committee of District Council of Physicians, Baśniowa 3, Warsaw (Resolution No. 45/05). Itraconazole is a drug with high intrasubject variability, and the formulation belongs to the group of high variability drug product (HVDP). Therefore, the majority of pharmacokinetic profiles began and ended at different time points (different time of absorption delay and concentration with values <LLOQ in last sampling points). For the transformation of data, the only C–T profiles that were chosen were those which originated from different subjects and those having identical number of indicated concentrations in the same interval. Ten C–T profiles were obtained this way between 1.5 and 48 h after the drug administration (Table 1).

**Table 1:** Concentrations of itraconazole administered orally at a single dose of 100 mg (Sporanox^®^ 100 mg tab., Janssen Pharmaceuticals) for 10 male subjects. Data before (1–48 h sampling points) and after transformation (1–36 h sampling points)

Time (h)	1	2	3	4	5	6	7	8	9	10
*Drug concentration before transformation (ng × ml* ^−1^ *)*										
1.5	8.77	8.76	11.84	3.44	6.54	4.44	3.76	4.70	3.02	17.75
2.0	36.03	15.42	17.89	8.33	24.53	6.21	11.01	48.30	5.50	31.86
2.5	70.67	75.36	31.76	13.67	29.50	8.47	26.35	85.58	10.99	68.10
3.0	64.63	80.38	22.58	20.96	47.81	15.37	30.01	76.89	13.12	66.20
3.5	55.60	69.68	22.67	36.48	38.25	20.94	29.80	60.41	18.65	57.94
4.0	57.98	61.19	31.00	40.29	55.28	33.00	32.17	77.47	24.26	53.52
4.5	57.06	62.77	42.74	15.36	61.97	33.91	43.94	77.47	24.13	48.26
5.0	47.82	56.86	35.50	45.25	47.74	37.70	64.72	80.07	32.21	44.12
6.0	38.96	39.49	28.50	29.79	32.54	37.12	54.68	71.13	33.49	24.79
8.0	31.71	25.14	16.31	22.55	30.31	27.75	32.54	42.86	26.56	18.42
12.0	24.04	19.78	11.54	12.63	16.18	15.34	21.72	31.69	18.83	13.02
24.0	12.26	11.92	5.11	5.86	7.17	7.40	10.36	11.18	4.62	6.15
36.0	6.23	4.54	5.05	3.41	3.97	4.02	5.62	7.41	5.18	4.10
48.0	3.13	5.26	3.39	3.14	3.17	3.64	3.22	4.59	3.18	2.92
*Drug concentration after transformation (ng × ml* ^−1^ *)*										
1.5	6.75	6.75	9.12	4.23	8.04	5.46	4.62	5.78	3.71	13.67
2.0	27.75	18.96	22.00	10.24	18.89	7.64	13.54	37.20	6.76	24.54
2.5	54.43	58.04	39.06	16.81	36.28	10.42	32.41	65.91	13.52	52.45
3.0	49.78	61.90	27.77	25.78	36.82	18.90	36.91	59.22	16.14	50.98
3.5	42.82	53.66	27.88	44.86	47.04	25.75	36.65	46.52	22.94	44.62
4.0	44.65	47.13	38.13	49.55	42.57	40.58	39.56	59.66	29.84	41.22
4.5	43.94	48.34	52.56	18.89	47.73	41.70	54.04	59.66	29.68	37.17
5.0	58.81	43.79	43.66	55.65	58.71	46.37	49.84	61.67	39.61	54.26
6.0	47.91	30.41	35.05	36.64	40.02	45.65	42.11	54.78	41.19	30.49
8.0	24.42	30.92	20.06	27.73	23.34	21.37	25.06	33.01	32.66	22.65
12.0	18.51	15.23	14.19	15.53	19.90	18.87	16.73	24.41	14.50	16.01
24.0	9.44	9.18	6.28	7.21	8.82	9.10	7.98	8.61	5.68	7.56
36.0	4.80	5.58	3.89	4.19	4.88	4.94	4.33	5.71	3.99	5.04
48.0^a^	3.13	5.26	3.39	3.14	3.17	3.64	3.22	4.59	3.18	2.92

^a^Data are not subject to transformation

### Assumptions

The source of variability in *C*_max_ point and for concentrations illustrating *C*_last_ are different and are the result of different processes, which are subject to the drug molecule in the two time points. Components that generate the *C*_max,CV %_ value are inter alia: variability resulting from the absorption process (CV %_,abs_), variability resulting from the distribution process (CV _%,dist_), variability resulting from the elimination process (CV %_el_) and CV %_an_. CV %_an_ which in this case is expressed by the precision of the method designated for the value equal to LLOQ. The main components that generate the *C*_last,CV %_ value are CV %_el_ and CV %_an_. Both CV %_el_ and CV %_an_ to a large extent influence the value of *C*_max,CV %_, as the drug elimination process is simultaneous to the processes of distribution and absorption. Therefore, it was assumed that

1 $$ C_{{{ \hbox{max} },{\text{CV \% }}}} \approx {\text{CV \% }}_{\text{abs}} {\text{ + CV \% }}_{\text{dist}} {\text{ + CV \% }}_{\text{el}} {\text{ + CV \% }}_{\text{an}} $$

while in the case of a point in the elimination phase:

2 $$ C_{{{ \hbox{max} },{\text{CV}}\,\% }} \approx {\text{CV}}\,\%_{\text{el}} + {\text{CV}}\,\%_{\text{an}} $$

subtracting Eq. 2 from Eq. 1 the value

3 $$ C_{{{\text{max,CV\,\% }}}} = {\text{CV\,\% }}_{\text{abs}} {\text{ + CV\,\% }}_{\text{dist}} $$

is obtained, which allows to observe the *C*_max_ value, without the factors responsible for the variability of the qualifying process and the analytical method.

Adopting the above assumptions, a scheme of data transformation was proposed, which is illustrated by the following example: the arithmetic mean (*M*_*A*_) for the sampling point equal to 1.5 h is 7.30 ng ml^−1^, concentration value (*C*_*n*_) for one of the subjects in the analyzed series is before the transformation 8.77 ng ml^−1^ ($C_{n} > M_{A}$); the lowest value of variability in the elimination phase is the value obtained for the sampling point in the 48th hour and equals to 21.26 % (*C*_last,CV %_); CV %_an_ for the LLOQ value is 7.06 %; *C*_max,CV %_ in the analyzed group is 30.82 % therefore

4 $$ X = C_{\text{last,CV\,\%}}-(C_{\text{last,CV\,\%}})\times {\text{CV\,\%}}_{\rm an}/100$$

which in the case of the analyzed point is 19.76 %,

5 $$ Y = C_{\text{max,CV\,\%}}-(C_{\text{max,CV\,\%}})\times {\text{CV\,\%}}_{\rm an}/100$$

which in the case of the analyzed point is 28.64 %,

6 $$ Y_{1} = (Y \times C_{n} )/100 $$

represents the percentage of concentration value before the transformation (*C*_*n*_) calculated with the value of the variability *C*_max,CV %_ reduced with CV %_an_, which in the case of the analyzed point gives the value of 2.51 ng **×** ml^−1^.

7 $$ X_{1} = (Y_{1} \times X)/100, $$

represents the percentage of value *Y*_1_ calculated with the value C_last,CV %_ reduced with CV _%,an,_ which in the case of the analyzed point gives the value of 0.496 ng **×** ml^−1^.

The value of *C*_*n*_ after the transformation of (*C*_*n*T_) is

8 $$ C_{{n{\text{T}}}} = C_{n} + (Y_{1} - X_{1} ), $$

if

9 $$ C_{n} < M_{A}\; {\text{and}}\;C_{{n{\text{T}}}} = C_{n} - (Y_{1} - X_{1} ) $$

if $C_{n} > M_{A}$. In the case of the analyzed concentration point $C_{n} > M_{A}$ therefore$C_{{n{\text{T}}}} = 8.77 - (2.51 - 0.496) \,$, which after transformation gives the concentration equal to $C_{{n{\text{T}}}} = 6.75$ ng **×** ml^−1^. The transformation of all of the concentration points was made in an analogous way, excluding the series of concentration, which were the source for C_last,CV %_.

In developed form, used formulas take the form:if $C_{n} < M_{A}$, then C_*n*T_ takes the value:

10 $$ \begin{gathered} C_{{n{\text{T}}}} = C_{\text{n}} + [(((C_{{{\text{max,CV\,\% }}}} - (C_{{{\text{max,CV\,\% }}}} \times {\text{CV\,\% }}_{\text{an}} )/100) \times C_{n} )/100) - ((((C_{{{\text{max,CV\,\% }}}} - (C_{{{ \hbox{max} },{\text{CV\,\% }}}} \times \\ {\text{CV\,\% }}_{\text{an}} )/100 \times C_{n} )/100) \times (C_{{{\text{last,CV\,\% }}}} - (C_{{{\text{last,CV\,\% }}}} \times {\text{CV}}\,\%_{\text{an}} )/100))/100)] \\ \end{gathered} $$

if $C_{n} > M_{A}$, then C_*n*T_ takes the value:

11 $$ \begin{gathered} C_{{n{\text{T}}}} = C_{n} - [(((C_{{{\text{max,CV \% }}}} - (C_{{{ \hbox{max} },{\text{CV \% }}}} \times {\text{CV \% }}_{\text{an}} )/100) \times C_{n} )/100) - ((((C_{{{\text{max,CV \% }}}} - (C_{{{\text{max,CV \% }}}} \times \\ {\text{CV \% }}_{\text{an}} )/100 \times C_{n} )/100) \times (C_{{{\text{last,CV \% }}}} - (C_{{{\text{last,CV \% }}}} \times {\text{CV }}\%_{\text{an}} )/100))/100)] \\ \end{gathered} $$

### Pharmacokinetics and statistical analysis

Non-compartmental modeling was used to estimate pharmacokinetic parameters of itraconazole. Pharmacokinetic calculations were performed with the use of Phoenix™ WinNonlin^®^ 6.3 (Certara L.P.). The area under the C–T curve (AUC) from time 0 to the last concentration time point and for infinity (AUC_0-tlast_; AUC_0-inf_) as well as area under first moment of concentration time curve (AUMC) from time 0 to the last concentration time point (AUMC_0-tlast_), were determined by the trapezoidal method. Mean residence time (MRT_0-tlast_) from time 0 to the last concentration time point was calculated using the standard formula ${\text{MRT}}_{{ 0 {\text{ - tlast}}}} {\text{ = AUMC}}_{{ 0 {\text{ - tlast}}}} / {\text{AUC}}_{{ 0 {\text{ - tlast}}}} .$ The elimination rate constant (*k*_el_) was determined by linear regression of the last three points on the C–T curve. In relation to calculations of *t*_1/2kel_ in the specified population it is recommended to conduct the analysis with the harmonic mean and the proper value of pseudo SD (Lam et al. 1985). This is due to the fact that in the case of C–T data, the data distribution is inclined according to the log-normal model. Thus, the geometric mean (M_G_) and the corresponding coefficient of variation are the factors of descriptive statistics for *t*_1/2kel,_ which are considered to be more appropriate than the arithmetic mean (*M*_*A*_) (Keene 1995; Senn 2002; Gad 2009). In relation to *t*_1/2kel_, the harmonic mean and the value of pseudo SD were calculated. In relation to the other parameters *M*_*A*_ and *M*_*G*_ were calculated. As tool for measurement of central tendency, median (*M*) and his standard deviation (SD_*M*_) were used. A statistical analysis of *M*_*A*_, *M*_*G*_, *M* and their SD (SD_*A*_; SD_*G*_; SD_*M*_) was performed using Microsoft Office Excel^®^ software. The percent of relative standard deviation (CV %) was calculated using formula ${\text {CV\,\%}}\;=\;{\text{SD}}/{\text{M}}\times{100}$ Raw and transformed data correlations were confirmed by sign test and all pharmacokinetics correlations were confirmed by student-*t* test. Differences with *P* < 0.05 were regarded as statistically significant.

## Results

**Fig. 1:**
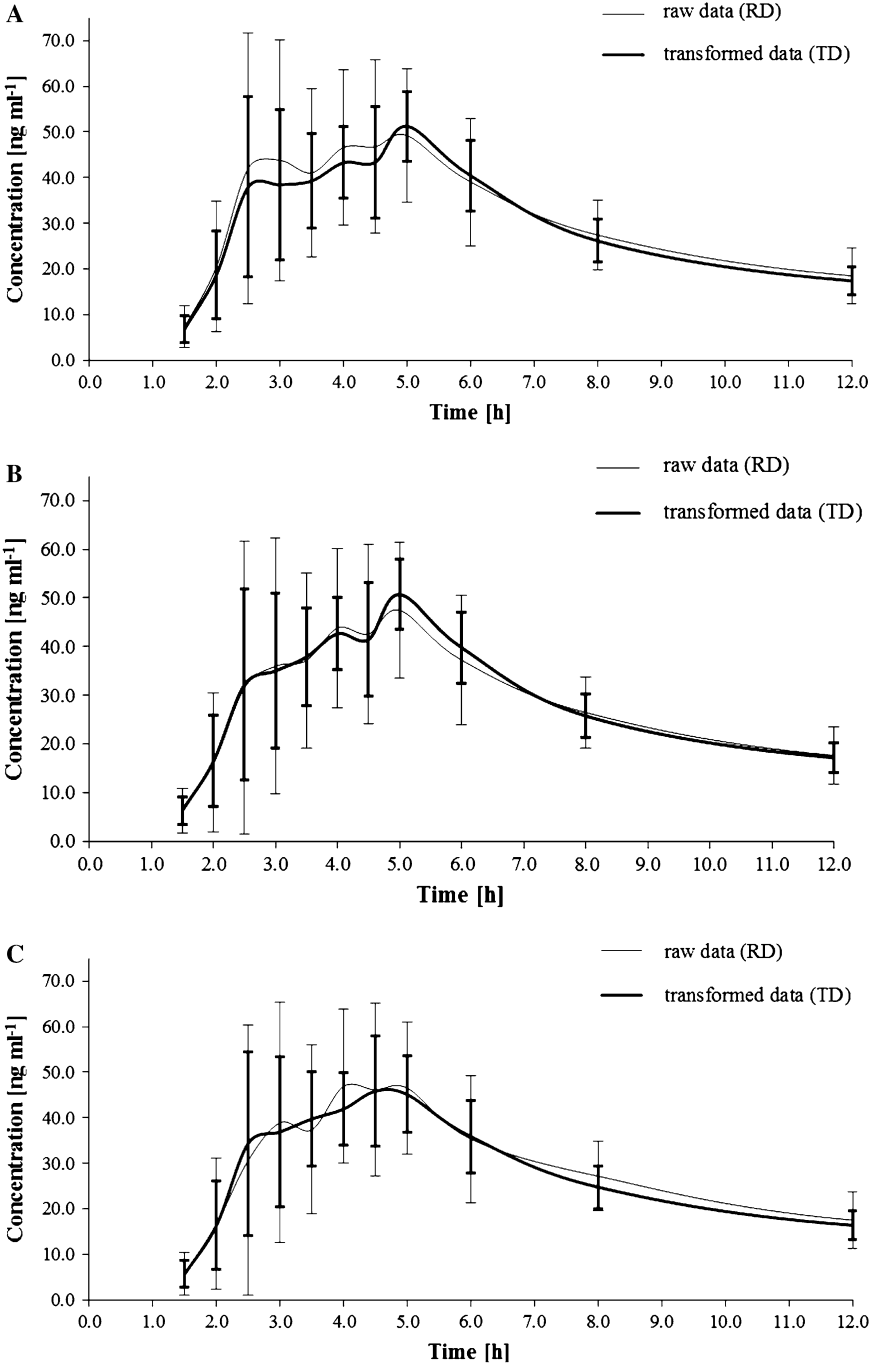
Concentration–time data of itraconazole administered orally at a single dose of 100 mg for 10 male subjects before (*solid line*) and after transformation (*dashed line*). **a** Represents arithmetic mean and standard deviation, **b** shows geometric mean and standard deviation and **c** shows median and standard deviation

The lowest values of CV % for raw data (RD) were noted for the sampling point 48 h after the administration of the drug, and the value was 20.61 % (Table 1). It was used to transform data in the rest of the time points (1.5–35 h). The concentration values after the transformation (TD) are presented in the Table 1. Image of differences between RD and TD for the largest fluctuation of C–T curve is presented in Fig. 1. On the basis of RD and TD, CV % was calculated for *M*_*A*_, SD_*A*_, *M*_*G*_, SD_*G*_, M and SD_*M*_, which is presented in the Table 2. In relation to the value of *M*_*A*_, *M*_*G*_ and *M* between the RD and TD data, there were no statistically significant differences (*P* > 0.05). No statistically significant differences (*P* > 0.05) were found between the mean values of individual points of concentration and the RD and TD data. The SD_*A*_ value and SD_*G*_ were significantly lower (*P* < 0.05) in relation to the RD group.

**Table 2:** Mean (*n* = 10), standard deviation, and relative standard deviation of raw data before and after transformation

Time (h)	Raw data (RD)									Transformed data (TD)								
	*M*_A_	SD_A_	RSD	*M*_G_	SD_G_	RSD	*M*	SD_M_	RSD	*M*_A_	SD_A_	RSD	*M*_G_	SD_G_	RSD	*M*	SD_M_	RSD
1.5	7.30	4.66	63.81	6.22	4.55	73.23	5.62	4.66	82.90	6.81	2.94	43.15	6.34	2.83	44.64	5.62	2.95	52.57
2.0	20.51	14.37	70.09	16.11	14.33	88.96	16.66	14.37	86.30	18.75	9.62	51.27	16.43	9.41	57.31	16.33	9.66	59.13
2.5	42.05	29.65	70.51	31.54	30.02	95.18	30.63	29.65	96.79	37.93	19.73	52.02	32.17	19.58	60.88	34.34	20.17	58.74
3.0	43.80	26.45	60.40	35.97	26.29	73.09	38.91	26.45	67.98	38.42	16.42	42.74	35.00	15.95	45.56	36.86	16.42	44.54
3.5	41.04	18.53	45.15	37.14	18.01	48.49	37.37	18.53	49.60	39.28	10.43	26.55	37.88	9.99	26.38	39.73	10.46	26.32
4.0	46.62	16.98	36.42	43.83	16.35	37.29	46.91	16.98	36.19	43.29	7.87	18.18	42.66	7.49	17.57	41.90	7.87	18.79
4.5	46.76	18.93	40.49	42.58	18.44	43.31	46.10	18.93	41.07	43.37	12.18	28.09	41.44	11.72	28.28	45.84	12.18	26.58
5.0	49.20	14.55	29.58	47.45	13.92	29.33	46.50	14.55	31.30	51.24	7.63	14.90	50.71	7.26	14.31	45.08	8.40	18.64
6.0	39.05	13.95	35.72	37.21	13.36	35.90	35.31	13.95	39.51	40.43	7.71	19.08	39.77	7.35	18.47	35.84	8.02	22.36
8.0	27.42	7.64	27.87	26.46	7.31	27.63	27.16	7.64	28.14	26.12	4.70	18.00	25.75	4.48	17.38	24.74	4.70	19.00
12.0	18.48	6.19	33.51	17.63	5.93	33.66	17.51	6.19	35.37	17.39	3.13	17.98	17.16	2.97	17.34	16.37	3.13	19.10
24.0	8.20	2.94	35.80	7.73	2.83	36.54	7.29	2.94	40.31	7.99	1.28	16.09	7.89	1.22	15.51	7.77	1.40	18.04
36.0	4.95	1.22	24.53	4.83	1.16	24.02	4.80	1.22	25.34	4.74	0.63	13.28	4.70	0.60	12.72	4.84	0.63	12.99
48.0^b^	3.56	0.76	21.26	3.50	0.72	20.61	3.20	0.76	23.68^a^	–	–	–	–	–	–	–	–	–
*P* value										>0.05	0.035	0.006	>0.05	0.035	0.009	>0.05	0.039	0.010

*P*-value between raw (1.5–36 h) and transformed data (1.5–36 h)

*M*_*A*_ arithmetic mean (ng **×** ml^−1^); *SD*_*A*_ standard deviation of M_A_, *M*_*G*_ geometric mean (ng **×** ml^−1^), *SD*_*G*_ standard deviation of M_G_, *M* median (ng **×** ml^−1^), *SD*_*M*_ standard deviation of M, *RSD* relative standard deviation (%)

^a^Value of *C*_last,CV %_

^b^Data which are not transformed

The results of the key calculations of pharmacokinetic parameters are shown in Table 3. The analysis of SD_*G*_ pharmacokinetic parameters in RD and TD groups indicated more than twice lower value of SD_*G*_ in TD group. The CV % (${\text{SD}}_{A} /M_{A} \times 100$) of pharmacokinetic parameters, such as *k*_el,_ *t*_1/2kel_, *t*_max_, *C*_max_, AUC_0-tlast_, AUC_0-inf_, AUMC_0-last_ and MRT_inf_ calculated on the basis of TD was lower by 21.25, 15.44, 17.43, 9.47, 9.85, 1.59, 10.39 and 2.69 %, respectively, than CV % obtained for the PK parameters in RD group but not statistically significant (*P* > 0.05). The CV % (${\text{SD}}_{G} /M_{G} \times 100$) of pharmacokinetic parameters *k*_el,_ *t*_1/2kel,_ *t*_max_, *C*_max,_ AUC_0-tlast,_ AUC_0-inf,_ AUMC_0-last_, and MRT_inf_ calculated on the basis of TD was significantly lower (*P* < 0.05) by 39.40, 30.75, 44.13, 59.42, 53.77, 51.82 and 38.83 %, respectively, from CV % obtained for the PK parameters in RD group. The ratio of *M*_*G*_ in the RD group to M_G_ in the TD group ranged between 0.935 and 1.041 and was not statistically significant (*P* > 0.05).

**Table 3:** Pharmacokinetics parameters of itraconazole (administered orally at a single dose of 100 mg for ten male subjects) calculated using data before and after transformation

Stat.	*k*_el_ (h^−1^)	*t*_1/2kel_ (h)	*t*_max_ (h)	*C*_max_ (ng × ml^−1^)	AUC_0-tlast_ (ng × h × ml^−1^)	AUC_0-inf_ (ng × h × ml^−1^)	AUMC_0-tlast_ (ng × h^2^ × ml^−1^)	MRT_0-tlast_ (h)
Pharmacokinetics analysis using raw concentrations (RD)								
M_A_	0.03	24.33^**H**^	4.05	59.06	632.48	755.78	8,916.26	14.21
SD_A_	0.01	10.51	1.30	18.20	184.64	171.38	2,336.14	0.89
RSD	38.56	43.19	32.11	30.82	29.19	22.68	26.20	6.25
M_G_	0.03	22.54	3.85	56.37	610.57	740.33	8,662.64	14.19
SD_G_	0.01	10.13	1.25	17.48	176.53	163.32	2,230.72	0.84
RSD	39.94	44.94	32.49	31.00	28.91	22.06	25.75	5.94
M	0.03	24.33	4.05	59.06	632.48	755.78	8,916.26	14.21
SD_M_	0.01	9.97	1.23	17.27	175.16	162.58	2,216.26	0.84
RSD	36.58	40.97	30.47	29.24	27.69	21.51	24.86	5.93
Pharmacokinetics analysis using transformed concentrations (TD)								
M_A_	0.03	20.48	4.55	54.94	607.90	705.07	8,610.97	14.19
SD_A_	0.01	8.67	1.21	15.33	159.98	157.34	2,021.77	0.86
RSD	29.62	42.32	26.52	27.90	26.32	22.32	23.48	6.08
M_G_	0.03	21.07	4.42	54.49	602.87	697.74	8,546.80	14.18
SD_G_	0.01	5.29	0.99	6.86	80.57	107.76	1,060.43	0.52
RSD	24.20	25.11	22.50	12.58	13.37	15.44	12.41	3.63
M	0.03	21.69	4.55	54.94	607.90	705.07	8,610.97	14.19
SD_M_	0.01	5.25	0.99	6.84	80.42	107.51	1,058.49	0.52
RSD	23.36	24.22	21.67	12.45	13.23	15.25	12.29	3.63

*H* harmonic mean, *M*_*A*_ arithmetic mean, *SD*_*A*_ standard deviation of M_A_, *M*_*G*_ geometric mean, *SD*_*G*_ standard deviation of M_G_, *M* median, *SD*_*M*_ standard deviation of M; *RSD* relative standard deviation (%), *k*_*el*_ elimination rate constant; *t*_*1/2kel*_ half life in elimination phase, *t*_*max*_ time to reach maximum concentration, *C*_*max*_ maximum concentration, *AUC*_*0-tlast*_ area under the curve between zero and last concentration, *AUC*_*0-inf*_ area under the curve from zero to infinity, *AUMC*_*0-last*_ area under the first moment curve between zero and last concentration, *MRT*_*inf*_ mean residence time (AUMC_0-tlast_/AUC_0-tlast_)

## Discussion

This manuscript presents the method of data transformation calculated on the basis of PK parameters expressed as *M*_*A*_, *M*_*G*_ and *M*. Until now, only a few methods of transforming the values of PK parameters were proposed (Abdallah 1998; Fujita et al. 2006). Frequently, these methods were based on data transformation through the normalization of pharmacokinetic parameters, value of the dose or physiological parameters (body weight, dose, body surface, normalization etc.), (Sathirakul et al. 2003; Sathyan et al. 2007; Staatz and Tett 2007). Normalization and scaling of pharmacokinetic data are also used in the allometric analysis, in scaling either concentrations, time or pharmacokinetic parameters (Mahmood 2005). The purpose of these methods is to facilitate comparative analysis in pharmacokinetics. These methods, however, do not use the variation values obtained in the study to transform data. In the C–T data sequences standard deviation in individual time points within the population is different and ranges from low to high. This is true for both, the analysis of the variability within and between subjects. In the analyzed case, the phase of itraconazole absorption is a subject of large fluctuations. After a single administration of the drug, the low values of deviation for individual C–T points usually fall at the last sampling points. In these points, usually equal to or a bit higher than the LLOQ value, the variability of the SD value often does not exceed 10 %. This occurs because these are the concentrations which usually represent only the process of elimination that is not affected by the factors responsible for the variability of the kinetics of absorption and distribution of the drug. In relation to a single administration, the exceptions are the cases of redistribution of the drug in the late elimination phase (Davis et al. 2000; Coldham et al. 2002; Chrenova et al. 2010). Also, in the analyzed case the volatility calculated for each sampling point was the lowest at a point closest to the LLOQ value of the analytical method. It happens differently in the case of concentrations analyzed in relation to oral administration, while the drug is still present in the stomach or the process of absorption has begun in the intestines. Depending on many factors, the variability in this phase can be very high (Duquesnoy et al. 1998; Tubic et al. 2006). After transforming the itraconazole data, lower variability of concentration data gives more selective pharmacokinetics profile in absorption and early distribution phase.

In summary, the proposed method allows to achieve a reliable picture of the pharmacokinetic profile, free of substantial interference in the average values of obtained concentrations and in the values of pharmacokinetic parameters with a simultaneous decrease in the value of SD. This makes it easier to evaluate the C–T data at points, in which the SD is particularly high. Reducing the value of SD for studies such as first in man, pilot or the rare ones, due to the difficulty of collecting sufficient number of subjects, can help to make a decision about the further direction of the research.
